# Hematological toxicity of anti-tumor antibody-drug conjugates: A retrospective pharmacovigilance study using the FDA adverse event reporting system

**DOI:** 10.1371/journal.pone.0334513

**Published:** 2025-10-27

**Authors:** Mei Rao, Lihua Wu, Hong Chen, Xiaohui Wu, Huiying Wang, Yangyi Chen, Chunmei Chen

**Affiliations:** 1 Department of Pharmacy, Longyan First Affiliated Hospital of Fujian Medical University, Longyan, Fujian, China; 2 The School of Pharmacy, Fujian Medical University, Fuzhou, Fujian, China; 3 Department of Obstetrical, Longyan First Affiliated Hospital of Fujian Medical University, Longyan, Fujian, China; 4 Department of Radiology, The Second Hospital of Longyan City, Longyan, Fujian, China; Icahn School of Medicine at Mount Sinai Department of Pharmacological Sciences, UNITED STATES OF AMERICA

## Abstract

**Background:**

Although antibody-drug conjugates (ADCs) have shown significant efficacy in cancer treatment, hematotoxicity remains a serious issue. This study aims to investigate the relationship between ADCs and hematological toxicity.

**Methods:**

Our study was conducted using data extracted from the U.S. Food and Drug Administration Adverse Events Reporting System (FAERS) from the third quarter of 2011 to the second quarter of 2024. We used four disproportionality analysis methods to measure risk signals. In addition, we analyzed the time-to-onset of hematotoxicity adverse events (AEs).

**Results:**

A total of 4,803 cases of hematotoxicity AEs associated with ADCs were identified, the median age of patients was 60 years (IQR: 47–72). Different ADCs have different hematotoxicity profiles, among which brentuximab vedotin (BV) and sacituzumab govitecan (SG) were more likely to lead to serious outcomes. The median time-to-onset of hematotoxicity AEs was the shortest for SG at 12 days and the longest for trastuzumab deruxtecan (TG) at 22 days. The hospitalization and mortality rates with hematotoxicity AEs were 30.38% and 18.30%, respectively.

**Conclusions:**

ADCs are significantly associated with increased reporting of hematotoxicity. A novel hematotoxicity signal that was not disclosed in the drug specifications was observed. The most prominent hematotoxicity AE signals were cytopenia related to inotuzumab ozogamicin (IO), polatuzumab vedotin (PV), loncastuximab tesirine (LT), and tisotumab vedotin (TV); febrile bone marrow aplasia related togemtuzumab ozogamicin (GO), BV, and SG; and myelosuppression related to BV, trastuzumab emtansine (TE), andenfortumab vedotin (EV). Our findings need to be validated by large-scale prospective studies.

## 1. Introduction

Antibody-drug conjugates (ADCs), a novel class of targeted anti-tumor drugs, combine highly specific monoclonal antibodies with potent cytotoxic drugs. ADCs specifically deliver cytotoxic payloads into cancer cells to improve therapeutic efficacy while reducing systemic toxicity [1,2]. Since the first ADCs gemtuzumab ozogamicin was approved for acute myeloid leukemia treatment in 2000, ADCs development has undergone three generations of technological innovation that significantly improved targeting precision and safety profiles [3,4]. Currently, more than 140 ADCs are in clinical trials, highlighting their strong anti-tumor potential and their growing importance in cancer treatment [5].

Although ADCs exhibit significant potential in improving survival outcomes in patients with tumors, the toxic effects of ADCs, including neurotoxicity, ophthalmotoxicity, dermatotoxicity, and hematotoxicity, have attracted widespread attention [6–9]. Adverse events (AEs) related to hematotoxicity were reported in clinical trials [10–12], and hematotoxicity AEs have risen with the increased ADCs use. Hematotoxicity, which can be life-threatening, is a serious adverse reaction to ADCs, impacting treatment cycles and markedly impairing quality of life. In a systematic review and meta-analysis of AEs associated with HER-2-targeted ADCs therapy, the most common high-level AEs were thrombocytopenia (8.37%; 95% CI, 7.75%–9.07%, τ = 0.71), anemia (6.49%; 95% CI, 5.86%–7.11%, τ = 1.06), and neutropenia (6.42%; 95% CI, 5.76%–7.04%, τ = 1.21) [13]. Other meta-analyses have reported similar results [14]. In the ASCENT clinical trial, hematological toxicities in the sacituzumab govitecan group included neutropenia (63%), anemia (34%), leucopenia (16%), thrombocytopenia (5%), and neutropenic fever (6%); the most common grade >3 treatment-related AEs were hematological toxicities, including neutropenia and leukopenia [15]. However, the current black box warnings and precautions for ADCs from the US Food and Drug Administration (FDA) only mention the risks of neutropenia, hemorrhage, thrombocytopenia, and myelosuppression; other potential hematological toxicities have not been described. Our knowledge of ADCs-related AEs primarily relies on clinical studies and case reports [16,17], which are often limited by insufficient sample sizes and short follow-up periods. These limitations hinder a comprehensive understanding of ADCs-related hematotoxicity in a real-world setting, particularly the differences in the hematological toxicities among different ADCs. Thus, an extensive and thorough analysis of ADCs-related hematotoxicity based on post-marketing real-world data is important to bridge this knowledge gap and improve patient safety.

U.S. Food and Drug Administration Adverse Events Reporting System (FAERS) is a large, publicly available pharmacovigilance database with thousands of AE reports. FAERS is widely recognized for its standardized data [18]. Researchers can uncover potential safety signals using FAERS data that may not be evident in clinical trials, thereby strengthening market regulations and enhancing drug safety. This study aimed to analyze the hematotoxicity associated with ADCs using the FAERS database. The primary objectives were to provide a scientific basis for the safety assessment and risk management of ADCs for antitumor therapies and to provide practical insights for future pharmacovigilance research and clinical practice. Through this comprehensive analysis, we aimed to enhance the understanding of ADCs-related hematotoxicity and contribute to safer therapeutic strategies for cancer patients.

## 2. Methods

### 2.1. Data resource

The FAERS database is an authorized public database for the post-marketing safety monitoring of drugs. The FAERS database includes worldwide self-initiated AE reports from healthcare professionals, individual patients, and pharmaceutical companies Thus, the FAERS database is an invaluable resource for monitoring medication safety [19]. The FAERS database is divided into seven data sub-tables [20]: the DEMO table presents basic demographic characteristics of patients; the DRUG table provides a detailed record of the drugs or biologics along with their dosages and other relevant information; the REAC table includes all reported adverse events; the OUTC table includes treatment outcomes following the occurrence of AEs; the RPSR table provides the sources of reports; the THER table gives the start and end dates of drug treatments; and the INDI provides the indications for the use of the reported drugs.

For this real-world retrospective pharmacovigilance study, data were collected from the FAERS database from the third quarter of 2011 (2011 Q3) to the second quarter of 2024 (2024 Q2). Data were evaluated using disproportionality analysis. The search for ADCs-related AEs yielded 18,157,112 reports, and the number was reduced to 15,864,338 reports after removing duplicates so only the most recent report for each patient was retained, based on the FDA’s recommendation.

### 2.2. Drug and AEs definitions

AE reports concerning FDA-approved ADCs, including gemtuzumab ozogamicin (GO), brentuximab vedotin (BV), trastuzumab emtansine (TE), inotuzumab ozogamicin (IO), polatuzumab vedotin (PV), enfortumab vedotin (EV), trastuzumab deruxtecan (TD), sacituzumab govitecan (SG), loncastuximab tesirine (LT), tisotumab vedotin (TV), and mirvetuximab soravtansine (MS), as primary suspects (PS) were extracted. Non-FDA-approved ADCs, including cetuximab sarotuximab sodium and decitabine vedotin, were not included due to underreporting in the FAERS database. Moxetumomab pasudotox and belantamab mafodotin are also excluded because they were withdrawn from the US market in 2023 and 2020, respectively. AEs in the FAERS database are divided into four role codes: PS (primary suspect), SS (secondary suspect), C (concomitant), and I (interacting). To ensure that the AEs were most likely due to ADCs use, only PS reports were included in the analysis [21].

The AEs in this study were classified using the preferred terms (PT) in the Medical Dictionary for Regulatory Activities (MedDRA) (version 27.0) within the FAERS database. MedDRA is organized according to the terminology hierarchy divided into lowest level term (LLT), PT, higher level term (HLT), higher level group term (HLGT), and systems and organ class (SOC) [22]. PTs in this study were in the “blood and lymphatic system disorders” SOC.

### 2.3. Data mining

**Fig 1:**
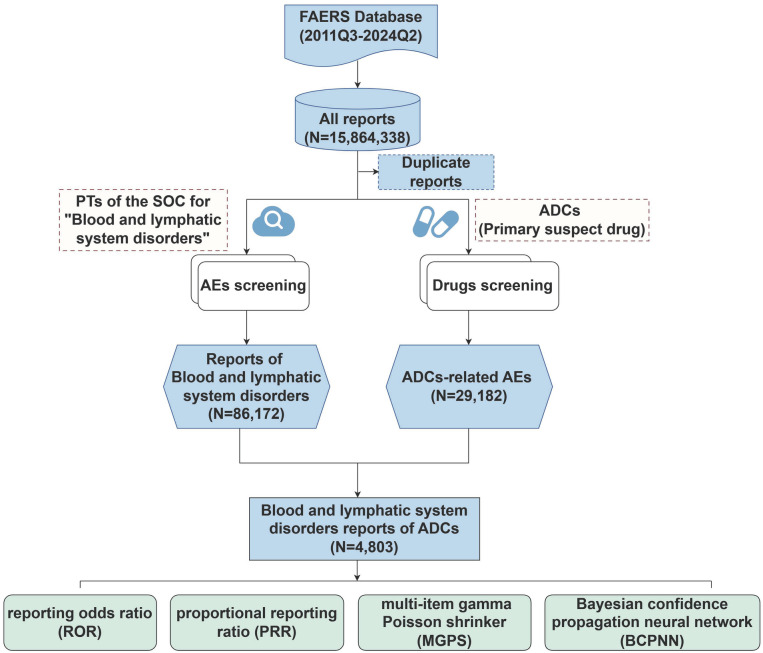
Flow chart of data queries for ADCs-related hematotoxicity AEs in the FAERS database. FAERS, FDA Adverse Event Reporting System; PT, preferred term; SOC, systems and organs class; AEs, adverse events; ADCs, antibody-drug conjugates.

Associations between ADCs and hematotoxicity AEs were investigated using a disproportionality methods. Disproportionality methods can only detect statistical signals or associations between drugs and adverse AEs but cannot establish causality. This method, which is commonly used for signal detection in pharmacovigilance studies, is based on a 2 × 2 contingency table (S1 Table). The disproportionality method aims to identify potential safety signals and assess the risk of drug-related AEs [23–25]. The reporting odds ratio (ROR) and proportional reporting ratio (PRR) are highly sensitive frequency methods, whereas the Bayesian confidence propagation neural network (BCPNN) and multi-item gamma Poisson shrinker (MGPS) methods are highly specific Bayesian methods [26]. In this study, the ROR and PRR methods were employed for signal mining, and the BCPNN and MGPS were applied to confirm the identified AE signals and reduce false positives [27,28]. The criteria and formulas for the four algorithms are shown in S2 Table. Larger algorithm values and stronger AE signals indicate stronger associations between ADCs and hematotoxicity AEs. Details of the data processing are shown in Fig 1.

### 2.4. Statistical analysis

The clinical characteristics of ADCs-related hematotoxicity AEs are demonstrated by descriptive analyses. ADCs-related hematotoxicity AEs in the serious and non-serious groups were compared using the chi-square and Fisher’s exact tests. Life-threatening, hospitalization, disability, and death were defined as serious AEs. In addition, the time-to-onset of ADCs-related hematotoxicity AEs was defined by the following equation: time-to-onset = start of drug administration (START_DT) − occurrence of adverse event (EVENT_DT). Incomplete or implausible time records were excluded from the time-to-onset analysis, including records showing AEs onset before the time of medication administration. The relationship between ADCs and hematotoxicity AEs occurrence intervals was analyzed using the Weibull shape parameter (WSP) test. The shape parameter, β, and 95% confidence interval (CI) were calculated. The shape parameter β of the WSP was used as the hazard rate. A hazard rate that decreased over time (early failure type) was defined as a shape parameter β < 1 and a 95% CI < 1. A constant hazard rate (random failure type) was defined as a shape parameter β close to or equal to 1, and a 95% CI that included 1. A hazard rate that increased over time (wear-out failure type) was defined as a shape parameter β > 1 and a 95% CI that included 1 [29]. All analyses were performed using R version 4.4.1, SPSS version 25.0, and Excel 2019.

## 3. Results

### 3.1. Summary of AEs reported in the FAERS database

After excluding duplicate reports, 15,864,338 records were extracted from the FAERS database; 86,172 reports were related to hematological toxicity. From these records, 29,182 AEs were suspected to be related to ADCs, and 4,803 reports identified ADCs as the primary suspected drugs of hematological toxicity. Hematotoxicity AEs accounted for 16.46% (4,803/29,182) of the total AEs reported for all the ADCs. The number of cases of hematotoxicity AEs increased significantly in recent years (Table 1). Notably, the incidence of hematotoxicity associated with ADCs increased. Higher rates of hematotoxicity AEs were observed with BV, TD, and SG [27.23% (1,308/4,803), 14.84% (713/4,803), and 14.76% (709/4,803), respectively]. MS, LT, and TV had relatively low rates of hematotoxicity AEs [0.35% (17/4,803), 0.40% (19/4,803), and 0.69% (33/4,803), respectively] (S3 Table).

**Table 1:** The clinical characteristics of cases from the FAERS database with ADCs-related hematotoxicity AEs from 2011Q3 to 2024Q2.

Clinical characteristics	Cases (N)	Proportion (%)
**Total cases (N)**	4,803	/
**Reporting year**		
** 2011Q3–2013**	158	3.29
** 2014–2016**	355	7.39
** 2017–2019**	546	11.37
** 2020–2022**	1722	35.85
** 2023–2024Q2**	2022	42.10
**Sex**		
** Female**	2,521	52.49
** Male**	1,604	33.40
** Unspecified**	678	14.12
**Weight (kg)**		
** <50 kg**	302	6.29
** 50 ≤ kg ≤ 100**	1563	32.54
** >100 kg**	115	2.39
** Unspecified**	2,823	58.78
**Age (years)**		
** Juvenile (<18)**	122	2.54
** Adult (18–65)**	1,699	35.37
** Seniors (≥ 65)**	1,311	27.30
** Unspecified**	1,671	34.79
**Median (IQR)**	60 (47–72)	/
**Reporting sources**		
** Physician**	3,160	65.79
** Pharmacist**	291	6.06
** Other healthcare providers**	884	18.41
** Customers**	440	9.16
** Unspecified**	28	0.58
**Reporting countries (Top ten)**		
** America**	1,034	21.53
** Japan**	1,071	22.30
** France**	516	10.74
** China**	411	8.56
** Germany**	264	5.50
** Canada**	207	4.31
** Italy**	112	2.33
** United Kingdom**	130	2.71
** Spain**	85	1.77
** Brazil**	78	1.62
** Others**	880	18.32
** Unspecified**	15	0.31
**Indications**		
** Lymphoma**	1,673	34.83
** Breast cancer**	1,424	29.65
** Bladder cancer**	473	9.85
** Leukemia**	327	6.81
** Gastric cancer**	127	2.64
** Others**	297	6.18
** Missing or unspecified**	482	10.04
**Suspected drugs**		
** Gemtuzumab Ozogamicin**	231	4.81
** Brentuximab Vedotin**	1,308	27.23
** Trastuzumab Emtansine**	553	11.51
** Inotuzumab Ozogamicin**	185	3.85
** Polatuzumab Vedotin**	487	10.14
** Enfortumab Vedotin**	548	11.41
** Trastuzumab Deruxtecan**	713	14.84
** Sacituzumab Govitecan**	709	14.76
** Loncastuximab Tesirine**	19	0.40
** Tisotumab Vedotin**	33	0.69
** Mirvetuximab Soravtansine**	17	0.35
**Outcomes**		
** Death**	879	18.30
** Life-threatening**	301	6.27
** Hospitalization**	1459	30.38
** Disability**	17	0.35
** Others**	1614	33.60
** Unspecified**	533	11.10

IQR, interquartile range; ADCs, antibody-drug conjugates.

### 3.2. Descriptive analysis of hematotoxicity AEs among ADCs users

Table 1 shows the clinical characteristics of ADCs-related hematotoxicity cases. After excluding data with unspecified sex information (678/4,803, 14.12%), the proportion of ADCs-related cases was higher in females (2,521/4,803, 52.49%) than in males (1,604/4,803, 33.40%). Adults (18–65 years) accounted for 35.37% (1,699/4,803) of the cases, followed by seniors who accounted for 27.30% of cases (> 65 years). Physicians (65.79%, 3,160/4,803) and other healthcare providers (18.41%, 884/4,803) were the main sources of the AE reports. Most reports concerning hematotoxicity AEs originated from Japan (22.30%, 1,071/4,803), America (21.53%, 1,034/4,803), and France (10.74%, 516/4,803). Lymphoma (34.83%, 1,673/4,803) and breast cancer (29.65%, 1,424/4,803) were the major indications; others accounted for 6.18% (297/4,803) of the cases, and missing or unspecified accounted for 10.04% (482/4,803) of the cases. The most common outcomes of hematotoxicity AEs were hospitalization (30.38%, 1,459/4,803) and death (18.30%, 879/4,803).

### 3.3. Disproportionality analysis for the association of hematotoxicity AEs with ADCs

The target AE signals were evaluated using the four algorithms listed in Table 2. GO, BV, TE, IO, PV, EV, TD, SG, and LT were associated with significantly higher reporting of hematotoxicity, and the results were consistent among the four algorithms. It should be noted that these associations are based on signals detected by spontaneous reporting systems and cannot be directly inferred as causal relationships. GO and PV exhibited the strongest association with hematotoxicity. Based on the ROR, PPR, and MGPS, TV may be related to hematotoxicity. None of the results for MS met the four algorithmic criteria; therefore, MS may not present a risk of hematotoxicity.

**Table 2:** Disproportionality analysis of all hematotoxicity associated with ADCs in the FAERS database.

Suspected drugs	Target ADCs		All other drugs		ROR (95% CI)	PRR (χ^2^)	EBGM (EBGM 05)	IC (IC 025)
	Event (a)	No event (b)	Event (c)	No event (d)				
**Gemtuzumab Ozogamicin**	295	1,985	767,643	45,714,954	8.85 (7.83–10.00)	7.83 (1787.73)	7.83 (7.07)	2.97 (1.30)
**Brentuximab Vedotin**	1,769	19,358	766,169	45,697,581	5.45 (5.19–5.72)	5.08 (5,876.83)	5.07 (4.87)	2.34 (0.68)
**Trastuzumab Emtansine**	682	12,229	767,256	45,704,710	3.32 (3.08–3.59)	3.20 (1,047.58)	3.20 (3.00)	1.68 (0.01)
**Inotuzumab Ozogamicin**	241	2,538	767,697	45,714,401	5.65 (4.95–6.45)	5.25 (843.00)	5.25 (4.70)	2.39 (0.73)
**Polatuzumab Vedotin**	729	5,231	767,209	45,711,708	8.30 (7.6–8.97)	7.41 (4,106.34)	7.40 (6.94)	2.89 (1.22)
**Enfortumab Vedotin**	679	9,832	767,259	45,707,107	4.11 (3.81–4.45)	3.91 (1,495.79)	3.91 (3.66)	1.97 (0.30)
**Trastuzumab Deruxtecan**	875	15,720	767,063	45,701,219	3.32 (3.10–3.55)	3.19 (1,339.45)	3.19 (3.01)	1.67 (0.01)
**Sacituzumab Govitecan**	959	10,614	766,979	45,706,325	5.38 (5.04–5.75)	5.02 (3,136.12)	5.02 (4.75)	2.33 (0.66)
**Loncastuximab Tesirine**	28	354	767,910	45,716,585	4.71 (3.2–6.92)	4.44 (75.80)	4.44 (3.22)	2.15 (0.48)
**Tisotumab Vedotin**	40	924	767,898	45,716,015	2.58 (1.88–3.54)	2.51 (37.01)	2.51 (1.93)	1.33 (−0.34)
**Mirvetuximab Soravtansine**	17	1,073	767,921	45,715,866	0.94 (0.58–1.52)	0.94 (0.06)	0.94 (0.63)	−0.08 (−1.75)
**In total**	6,314	79,858	761,624	45,637,081	4.74 (4.62–4.86)	4.46 (17,114.05)	4.44 (4.34)	2.15 (0.48)

ADCs, antibody-drug conjugates; ROR, reporting odds ratio; CI, confidence interval; PRR, proportional reporting ratio; EBGM, empirical Bayesian geometric mean; IC, information component.

Separate subgroup analyses based on age and sex (S1 Fig) using the lower limit of the 95% CI of EBGM revealed that the hematotoxicity risk signals for BV, EV, GO, IO, and PV were observed in all age and sex subgroups, and hematotoxicity risk signals for TD, LT, TE, SG, and TV were observed in most age and sex groups. The hematotoxicity risk signal for MS was not observed in any of the age and sex subgroups. The risk of hematotoxicity AEs in women was higher than that in men, and the risk of hematotoxicity AEs in adults (18–65 years) was higher than that in other age subgroups.

At the PT level, 135 positive signals for ADC-related hematotoxicity AEs were identified, involving 58 PTs. However, only 9 PTs are mentioned on the ADCs labels. The relevant PTs and signal values are listed in S4 Table. According to the BCPNN values, 15 signals (IC range: 2.09–8.17), 27 signals (IC range: 1.81–6.20), 11 signals (IC range: 1.26–7.91), 18 signals (IC range: 2.10–6.05), 19 signals (IC range: 1.96–5.73), 10 signals (IC range: 1.73–5.26), 12 signals (IC range: 1.71–5.93), 13 signals (IC range: 1.83–5.17), 5 signals (IC range: 2.32–3.71), 4 signals (IC range: 2.49–3.76) and 1 signal (IC = 2.26) were detected for GO, BV, TE, IO, PV, EV, TD, SG, LT, TV, and MS, respectively. HLT was used to classify positive signals for hematotoxicity AEs. Overall, there were 6, 6, 4, 7, 8, 4, 6, 5, 5, 3, and 1 positive signals for HLT level signals for GO, BV, TE, IO, PV, EV, TD, SG, LT, TV, and MS, respectively (S2 Fig).

**Fig 2:**
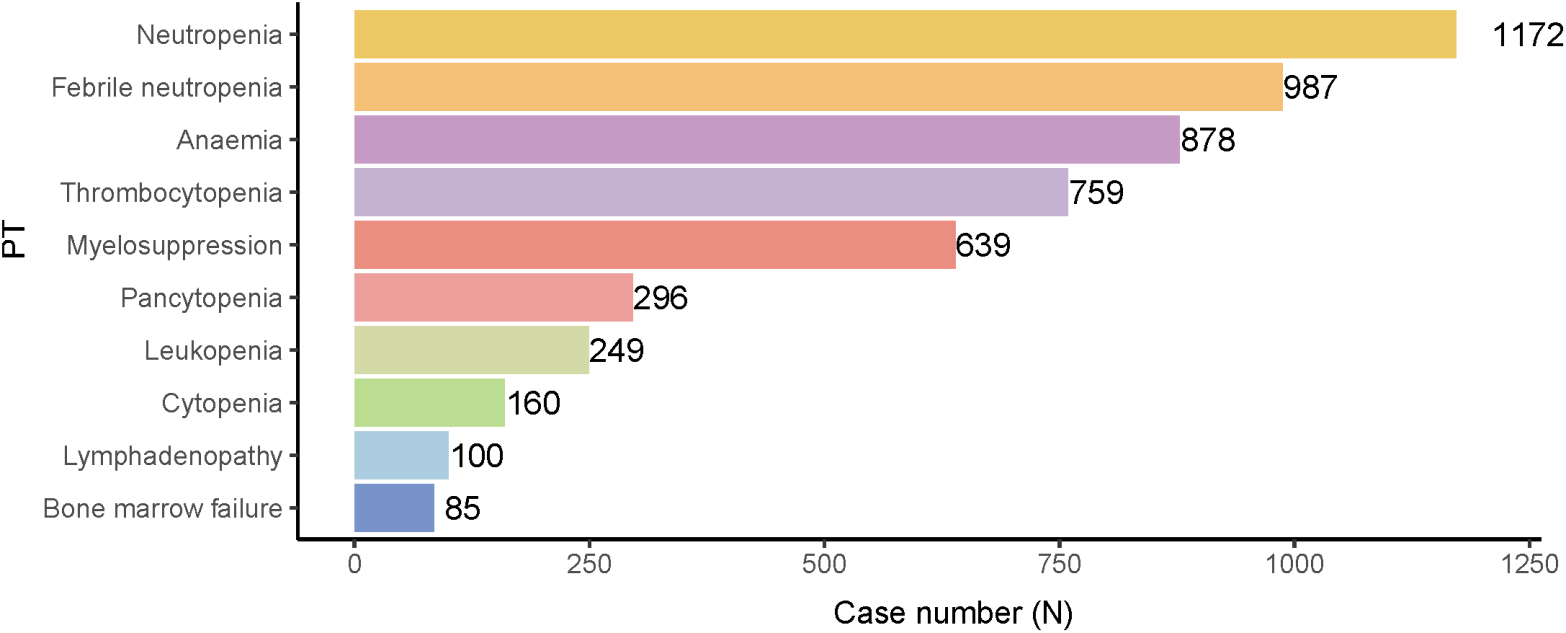
Number of reported cases for the most common ADCs-related hematotoxicity AEs categories according to different ADCs treatment regimens. ADCs, antibody-drug conjugates; AEs, adverse events; PT, preferred terms.

The top 10 most prominent hematotoxicity AEs following treatment with ADCs were neutropenia, febrile neutropenia, anemia, thrombocytopenia, myelosuppression, pancytopenia, leukopenia, cytopenia, lymphadenopathy, and bone marrow failure (S5 Table, Fig 2).

### 3.4. Time-to-onset of hematotoxicity AEs

**Fig 3:**
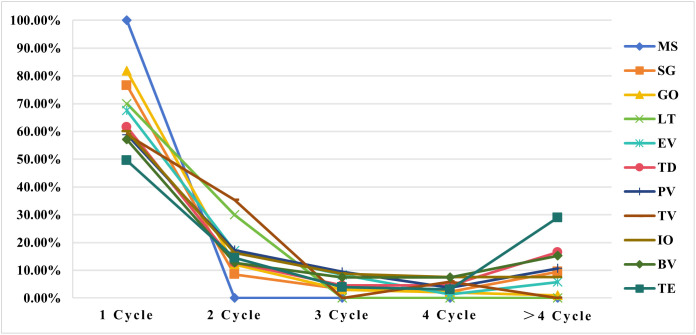
Time-to-onset of hematotoxicity AEs in individual case reports in the FAERS database. FAERS, FDA Adverse Event Reporting System.

Of the 4,803 AE reports of ADCs-related hematotoxicity, only 2,231 reports included a complete record of the time-to-onset. The onset times of the ADCs are summarized in Fig 3. The median onset time for SG of 12 days (IQR: 7–21 days) was the shortest. The onset time for TE of 22 days (IQR: 6–114.25 days) was the longest, and TE also had the widest onset time spread. The onset time range for GO was the narrowest, with a median of 13 days (IQR: 7–23 days). The median onset times for BV, IO, PV, EV, TD, LT, and TV were 14 days (IQR: 8–59 days), 14 days (IQR: 5–51.5 days), 13 days (IQR: 5–40 days), 14 days (IQR: 7–28 days), 15.5 days (IQR: 7–49.25 days), 16 days (IQR: 12.75–24.75 days), and 19 days (IQR: 9.5–30.5 days), respectively. The WSP test results for ADCs-related hematological toxicities are listed in Table 3. MS was not analyzed because the amount of data was too small. Most hematotoxicity AEs appeared within 30 days after the administration of ADCs. These results provide novel and useful insights for evaluating potential hematological toxicity risks of ADCs in the clinic.

**Table 3:** Weibull shape parameter tests and occurrence times for hematological toxicity associated with ADCs.

Suspected drugs	Case reports (N)	Median (IQR) (days)	Scale parameter: α (95% CI)	Shape parameter: β (95% CI)	Type
**Gemtuzumab Ozogamicin**	99	13 (7–23)	19.28 (15.40–23.17)	1.03 (0.89–1.18)	Random failure
**Brentuximab Vedotin**	683	14 (8–59)	37.62 (33.69–41.56)	0.76 (0.72–0.80)	Early failure
**Trastuzumab Emtansine**	228	22 (6–114.25)	64.23 (48.41–80.05)	0.56 (0.51–0.61)	Early failure
**Inotuzumab Ozogamicin**	80	14 (5–51.5)	30.25 (20.35–40.15)	0.71 (0.59–0.83)	Early failure
**Polatuzumab Vedotin**	243	13 (5–40)	26.31 (21.66–30.97)	0.75 (0.68–0.82)	Early failure
**Enfortumab Vedotin**	311	14 (7–28)	25.13 (21.75–28.51)	0.88 (0.81–0.95)	Early failure
**Trastuzumab Deruxtecan**	242	15.5 (7–49.25)	39.81 (31.87–47.75)	0.67 (0.61–0.73)	Early failure
**Sacituzumab Govitecan**	316	12 (7–21)	28.47 (23.44–33.50)	0.66 (0.62–0.71)	Early failure
**Loncastuximab Tesirine**	10	16 (12.75–24.75)	21.79 (14.80–28.80)	2.04 (1.09–2.99)	Wear-out failure
**Tisotumab Vedotin**	17	19 (9.5–30.5)	24.74 (15.82–33.66)	1.39 (0.88–1.90)	Random failure
**Mirvetuximab Soravtansine**	2	/	/	/	/

ADCs, antibody-drug conjugates; N, number of cases; IQR, interquartile range; CI, confidence interval.

### 3.5. Comparison of hematotoxicity profiles between the severe and non-severe groups

More than 55.30% of cases of ADCs-related hematotoxicity AEs resulted in serious consequences. Males (χ2 = 35.57, p < 0.0001), indication for gastric cancer (χ2 = 177.24, p < 0.0001), and BV (χ2 = 237.74, p < 0.0001) were associated with serious hematotoxicity AEs (Table 4). No significant differences in age (χ2 = 3.16, p = 0.206) or weight (χ2 = 4.45, p = 0.108) were detected between the two groups.

**Table 4:** Differences in clinical characteristics between cases of serious and non-serious ADCs-related hematotoxicity AEs.

Clinical characteristics	Total cases (N)	Serious cases (N, %)	Non-serious cases (N, %)	χ^2^	*p*-value
**Sex**				35.57, 1	< 0.001
** Female**	2,521	1,324 (52.52%)	1,197 (47.48%)		
** Male**	1,604	994 (61.97%)	610 (38.03%)		
** Unspecified**	678	338 (49.85%)	340 (50.15%)		
**Weight (kg)**				4.45, 2	0.108
** < 50 kg**	302	206 (68.21%)	96 (31.78%)		
** 50 ≤ kg ≤ 100**	1,563	1,036 (66.28%)	33.72%)		
** > 100 kg**	115	87 (75.65%)	28 (24.35%)		
** Unspecified**	2,823	1,327 (47.01%)	1,496 (52.99%)		
**Age (years)**				3.16, 2	0.206
** Juvenile (< 18)**	122	84 (68.85%)	38 (31.15%)		
** Adult (18–65)**	1,699	1,032 (60.74%)	667 (39.26%)		
** Seniors (≥ 65)**	1,311	805 (61.40%)	506 (38.60%)		
** Unspecified**	1,671	735 (43.99%)	936 (56.01%)		
** Median (IQR)**	60 (47–72)	60 (47–72)	60 (47–71)		
**Reporting sources**				17.50, 3	0.001
** Physician**	3,160	1,810 (57.28%)	1,350 (42.72%)		
** Pharmacist**	291	143 (49.14%)	148 (50.86%)		
** Other healthcare providers**	884	463 (52.38%)	421 (47.62%)		
** Customers**	440	220 (50.00%)	220 (50.00%)		
** Unspecified**	28	20 (71.43%)	8 (28.57%)		
** Reporting countries (Top ten)**				102.56,10	< 0.001
** America**	1,034	517 (50.00%)	517 (50.00%)		
** Japan**	1,071	578 (53.97%)	493 (46.03%)		
** France**	516	318 (61.63%)	198 (38.37%)		
** China**	411	196 (47.69%)	215 (52.31%)		
** Germany**	264	203 (76.89%)	61 (23.11%)		
** Canada**	207	96 (46.38%)	111 (53.62%)		
** Italy**	112	57 (50.89%)	55 (49.11%)		
** United Kingdom**	130	80 (61.54%)	50 (38.46%)		
** Spain**	85	63 (74.12%)	22 (25.88%)		
** Brazil**	78	46 (58.97%)	32 (41.03%)		
** Others**	880	491 (55.80%)	389 (44.20%)		
** Unspecified**	15	11 (73.33%)	4 (26.67%)		
**Indications**				177.24, 5	< 0.001
** Lymphoma**	1,673	1,086 (64.91%)	587 (35.09%)		
** Breast cancer**	1,424	646 (45.37%)	778 (54.63%)		
** Bladder cancer**	473	236 (49.89%)	237 (50.11%)		
** Leukaemia**	327	226 (69.11%)	101 (30.89%)		
** Gastric cancer**	127	100 (78.74%)	27 (21.26%)		
** Others**	297	181 (60.94%)	116 (30.06%)		
** Missing or unspecified**	482	181 (37.55%)	301 (62.45%)		
**Suspected drugs**				237.74, 10	< 0.001
** Gemtuzumab Ozogamicin**	231	153 (66.23%)	78 (33.77%)		
** Brentuximab Vedotin**	1,308	915 (69.95%)	393 (30.05%)		
** Trastuzumab Emtansine**	553	219 (39.60%)	334 (60.40%)		
** Inotuzumab Ozogamicin**	185	114 (61.62%)	71 (38.38%)		
** Polatuzumab Vedotin**	487	238 (48.87%)	249 (51.13%)		
** Enfortumab Vedotin**	548	256 (46.72%)	292 (53.28%)		
** Trastuzumab Deruxtecan**	713	341 (47.83%)	372 (52.17%)		
** Sacituzumab Govitecan**	709	386 (54.44%)	323 (45.56%)		
** Loncastuximab Tesirine**	19	11 (57.89%)	8 (42.11%)		
** Tisotumab Vedotin**	33	21 (63.64%)	12 (36.36%)		
** Mirvetuximab Soravtansine**	17	2 (11.76%)	15 (88.24%)		
** Total cases**	4,803	2,656	2,147		

ADCs, antibody-drug conjugates; N, number of cases; IQR, interquartile range; χ2, chi-square.

### 3.6. Fatality and hospitalization rates related with hematotoxicity AEs

**Fig 4:**
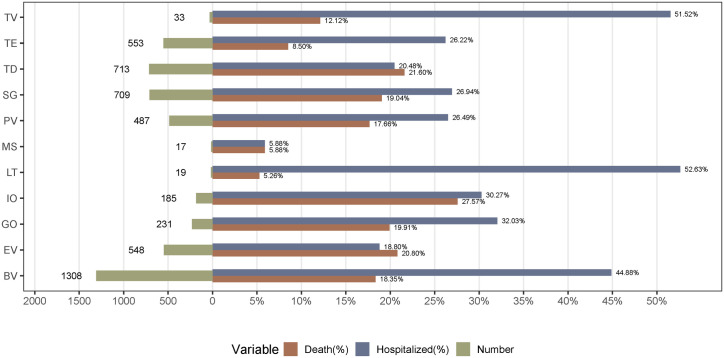
Number of reports and rates of death and hospitalization for ADCs-related hematotoxicity AEs. ADCs, antibody-drug conjugates.

The most severe outcome in each case was selected, and mortality and hospitalization rates were assessed to determine the prognosis for patients who experienced ADCs-related hematotoxicity AEs. As shown in Fig 4, the occurrence of hematotoxicity AEs was associated with poor prognosis; death and hospitalization occurred in 18.30% (879/4,803) and 30.38% (1,459/4,803) of the cases, respectively. The fatality rate was the highest for IO (27.57%), followed by TD (21.60%), EV (20.80%), GO (19.91%), SG (19.04%), BV (18.35%), PV (17.66%), TV (12.12%), TE (8.50%), MS (5.88%), and LT (5.26%). Interestingly, high hospitalization rates were not associated with high mortality rates. The hospitalization rate was the highest for hematotoxicity related to LT (52.63%), followed by TV (51.52%) and BV (44.88%).

## 4. Discussion

Recently developed ADCs in the field of anti-cancer drugs have provided new treatment options for patients. However, the clinical use of ADCs is accompanied by adverse reactions, including hematological toxicity. Effective management and safety assessment of potential hematological toxicity are important for both clinicians and patients. To the best of our knowledge, this is the first study to examine the correlation between ADCs and hematological toxicity in the real world using the FAERS database. The findings will help clinicians gain a more comprehensive understanding of the hematological toxicity profiles and time-to-onset characteristics of different ADCs, providing new insights and guidance for clinical treatment.

We noted an upward trend in the reported number of ADCs-related hematotoxicity during the period from 2020 to 2024Q2, which may be the result of multiple factors. Although the increased clinical application of ADCs and increased awareness of drug adverse reaction monitoring among healthcare system staff may be part of the reason, other factors may lead to reporting bias that is unrelated to the potential risk of actual hematotoxicity. For example, the reported number of cases may be exaggerated for drugs that have been recently approved or have received media attention. Strengthened regulatory measures by regulatory agencies may increase the reporting sensitivity of medical institutions. Therefore, the risk signals found in this study, especially for newer drugs or drugs with recent safety alerts, should be understood as being at least partially influenced by reporting bias rather than simply indicating a higher real risk.

In this study, 4,803 cases of hematotoxicity associated with ADCs were retrieved from the FAERS database, and ADCs-related hematotoxicity were described in terms of risk signals. Overall, the use of ADCs was significantly associated with hematotoxicity; However, MS was not significantly associated with hematotoxicity. This may be related to the shorter time on the market (November 2022 launch) and the fewer number of patients treated with MS. The incidence of hematotoxicity was higher in female compared with male. This sex difference may be due to the use of ADCs for the treatment of female-prone tumors such as breast and ovarian cancer, which accounted for 37.1% of all drug indications. Physiological factors may also play a role; men usually have higher hemoglobin levels than women, and hemoglobin levels in women may be influenced by blood loss during menstruation, sex hormone differences, and inadequate dietary iron intake in women [30]. Thus, the hematological system of female may be more sensitive to ADCs treatment. The highest number of hematotoxicity AEs was reported in adults (18–65 years). This discrepancy may be related to the selection bias of the treatment modalities. In addition, we were unable to count the exact incidence of hematotoxicity AEs in the ADCs-treated population due to the self-limiting nature of the FAERS database. Systematic reviews and meta-analyses that provide insight into the influence of sex and age differences on hematotoxicity effects are sparse. Given this research gap, further studies are needed to determine if sex and age should be considered by clinicians when administering ADCs treatment. Drug efficacy can be maximized and AEs can be minimized by providing personalized treatment strategies for different populations based on the risk factors for ADCs-related hematotoxic effects.

Hematological toxicity can occur at any time during drug administration. The onset of ADCs-related hematotoxicity occurred predominantly during the first two dosing cycles. The WSP tests revealed that TE, IO, PV, EV, SG, BV, and TD meet the characteristics of the early failure type, LT exhibits the characteristics of the wear failure type, and GO and TV belong to the random failure type. Based on these results, patients receiving TE, IO, PV, EV, SG, BV, or TD should be monitored for hematotoxicity during the first 14 days, and patients receiving LT should be monitored for hematotoxicity after one week. Patients receiving GO and TV should be monitored for hematotoxicity during the entire dosing cycle. The results of this study help clinicians identify high-risk populations, ages, and onset times for hematotoxicity associated with ADCs, enabling more effective management of ADCs-related hematotoxicity.

Neutropenia, febrile neutropenia, anemia, thrombocytopenia, myelosuppression, pancytopenia, leukopenia, hemocytopenia, lymphadenopathy, and bone marrow failure are the most likely AEs to occur in response to ADCs treatment. Different ADCs showed signals for different types of hematotoxicity AEs. SG, PV, LT, BV, TD, and EV, in that order, exhibited the highest risk of neutropenia. A meta-analysis demonstrated that the incidence of neutropenia after ADCs use was as high as 43.7% [31]. A clinical trial involving 235 patients with metastatic triple-negative breast cancer treated with SG showed that [32] neutropenia of any grade occurred in up to 63% of patients, 51% of patients reported > grade 3 or higher neutropenia, anemia of any grade occurred in 34% of patients, and leukopenia occurred in 10% of patients. Schlam et al [33] highlighted the hematological toxicity of SG; neutropenia occurred in up to 72% of cases, often necessitating dose reductions or dosing delays. The black box warning states that SG can lead to severe and even life-threatening neutropenia and recommends immediate anti-infective treatment for patients presenting with febrile neutropenia. PV combined with bendamustine and rituximab for treating relapsed/refractory diffuse large B-cell lymphoma resulted in grade 3–4 neutropenia in 29% of patients, thrombocytopenia in 38% of patients, and anemia in 19% of cases [34]. A review of LT noted that the most common grade >3 AEs were neutropenia and thrombocytopenia [35]. Zhang et al. reported that 6% of patients with metastatic uroepithelial cancer treated with EV experienced grade 3–4 neutropenia [36]. Our results are consistent with those of earlier studies. The ADCs with the highest risk of thrombocytopenia were TE, LT, GO, IO, PV, and MS, from the highest to lowest risk. In breast cancer clinical trials, patients treated with TE had a significantly higher rate of grade >3 thrombocytopenia than patients treated with TD [37–40]. Another study comparing the efficacy and safety of TD versus TE in patients with breast cancer showed that [41] the incidence of grade >3 drug-related thrombocytopenia was significantly higher in the TE group (34.6%) than in the TD group (12.9%); however, the incidence of neutropenia was more common in the TD group (25.9%). These studies highlight the differences in the hematological toxicity profiles between TD and TE. In a phase II clinical study of IO for the treatment of non-Hodgkin’s lymphoma, thrombocytopenia occurred in 77% of patients, and more than half of the patients were diagnosed with grade 3 toxicity [42]. In the LFA-0701 trial, the main toxicity of GO was long-term thrombocytopenia [43]. In the present study, PV, TV, LT, EV, TD, and TE were associated with a higher risk of anemia. Overall, our results and those of previous studies suggest that the major hematotoxicity profiles of ADCs are different. Only the black-box warning for SG showed significant haematotoxicity AEs. In addition, TE, TD, TV, GO, BV, IO, PV, and LT listed hematotoxicity AEs as a cautionary note in their inserts. Most of these warnings are related to hemorrhage, thrombocytopenia, neutropenia, and myelosuppression. Hematotoxicity AEs that were not mentioned in the existing ADCs drug inserts (S3 Table) were also detected. Thus, these results provide more comprehensive and detailed information on drug reactions for the clinical use of ADCs. These new AE signals suggest that unrecognized hematotoxicity AEs are lurking in approved ADCs. These hematotoxicity risks may be overlooked during clinical diagnosis and treatment, delaying diagnosis and treatment and possibly leading to irreversible and serious consequences. Therefore, medical staff should pay attention to and routinely monitor hematological indices after ADCs administration to ensure that hematotoxicity AEs are identified promptly and appropriately managed to improve therapeutic safety and efficacy.

Our findings suggest that ADCs have a potential risk of hematological toxicity, such as reduced leukopoiesis, platelet count, and erythropoiesis, similar to chemotherapy and conventional targeted anticancer drugs. The ADCs toxicity is mainly attributed to the off-target effects of the cytotoxic payloads [44]. The most common non-target-dependent dose-limiting toxicity of ADCs with auristatins, spicosporins, and medanserins as the cytotoxic “warheads” is hematological toxicity, and ADCs with the same payload or linker have highly similar hematotoxicity profiles [45]. In this study, more patients treated with microtubule polymerization inhibitor-ADCs (N = 2946) developed hematotoxicity than patients treated with DNA-damaging agent-ADCs (N = 1857). This may be because microtubule protein inhibitor-type ADCs have a higher risk. GO had the highest risk of hematotoxicity, followed by PV, IO, BV, SG, LT, EV, TD, TE, and TV, which had different cytotoxic payloads. The cytotoxic payloads of GO and IO are DNA-damaging galectin derivatives, and the payload of BV, PV, EV, and TV is the microtubulin inhibitor monolayer auristatin E (MMAE). The TE payload is the microtubulin inhibitor derivative maytansine DM1, the TD and SG payloads are topoisomerase I inhibitors, the LT payload is the alkylating agent pyrrolobenzodiazepine dimer, and the MS payload is a microtubule protein inhibitor, maytansinoid derivative DM4 [46,47]. The risk signals for GO and PV were significantly higher than the risk signals for the other ADCs. The differences in risk signals were associated with the toxicity of cytotoxic payloads and other components of ADCs [31].

The occurrence of serious outcomes, including hospitalization and death, is concerning. The overall hospitalization and mortality rates for patients who experience ADCs-related hematotoxicity were high (30.38% and 18.30%, respectively). Reports of ADCs-related severe hematotoxicity AEs were more common in male than in female (61.97% vs. 52.52%, p < 0.001). Although women reported a higher number of hematotoxicity AEs, men had a higher proportion of severe outcomes. More female reported hematotoxicity AEs may partly reflect the large number of breast cancer patients, long treatment cycle, more frequent monitoring and increased reporting opportunities [48]. However, lymphoma (especially Hodgkin’s lymphoma) has a higher incidence in young men [49]. The more severe outcome in men may be related to the invasiveness of lymphoma itself [50] and the stronger toxicity of drugs commonly used for lymphoma, such as BV and PV [51]. The observed sex differences are essentially a mixed reflection of the differences in indications and differences in drug toxicity characteristics. In this study, no significant differences in severe hematotoxicity AEs between age groups were detected, but ADCs-related severe hematotoxicity AEs were more common in patients younger than 18 years and older than 65 years. Therefore, we did not identify age as a significant risk factor for ADCs-related severe hematotoxicity. We did not identify body weight as a risk factor for ADCs-related severe hematotoxicity. The mortality rates due to hematotoxicity of different ADCs regimens were assessed and compared. The lethality rate (27.57%) for IO-related hematotoxicity was relatively high, whereas LT-related hematotoxicity exhibited a relatively low lethality rate (5.26%). IO is a rescue therapy for patients with relapsed or refractory CD22-positive precursor B-cell acute lymphoblastic leukemia (R/R CD22-positive BCP-ALL). A meta-analysis demonstrated that thrombocytopenia and neutropenia were the most common AEs during IO treatment and one of the most common causes of treatment-related deaths or early trial terminations [52]. The most common grade 3/4 AEs occurring in patients treated with the IO regimen in combination with bosutinib were thrombocytopenia (60%) and neutropenia (38%) [53]. In severe cases, these adverse reactions can increase the risk of bleeding and infection and become life threatening.

The target toxicity of the monoclonal antibodies is also an important factor in the toxicity level. CD33 antibodies in GO may bind to differentiated pluripotent hematopoietic cells expressing CD33 antigen, leading to significant hematological side effects [54,55]. GO also employs linkers with acid cleavage bonds (e.g., hydrazone), and instability of the linkers may cause premature release of the payload (calicheamicin) into the bloodstream, leading to off-target toxicity of ADCs [56]. For PV, hematotoxicity and off-targeting of the payload MMAE act directly on bone marrow hematopoietic cells, resulting in reduced neutrophil production [57]. This toxic effect is associated with most MMAE-ADCs that employ valine-citrulline as a linker (e.g., BV, EV, and TV) [56]. In addition, the SG payload SN-38 accelerates apoptosis in hematopoietic progenitor and stem cells [58]. The TE payload T-DM1 inhibits megakaryocyte differentiation through megakaryocytosis-mediated internalization, resulting in thrombocytopenia [59]. However, other mechanisms may also contribute to the hematotoxicity of ADCs. Therefore, the full mechanisms of ADCs-related hematological toxicity require further study.

Our study has some limitations. Firstly, as a retrospective study of a spontaneous reporting system, FAERS has inherent flaws such as reporting bias and incomplete information. In particular, the DRUG file often lacks actual drug records for patients, making it difficult to assess the impact of concomitant medications (such as ADCs and traditional chemotherapy drugs [60]) on hematological toxicity. Second, owing to the lack of detailed clinical data, we were unable to accurately distinguish whether adverse reactions were caused by drug interactions, disease progression, or other complications. Thirdly, the FAERS database may contain duplicate reports and missing data. Although we performed rigorous data cleaning and deduplication, this may have affected the accuracy of the results. Fourthly, some ADCs have fewer reported cases, which may increase the risk of false positive signals. Fifthly, the risk signals observed through the proportional imbalance analysis only suggest an association between ADCs and hematotoxicity but do not establish a causal relationship. Causal relationships should be assessed and validated through rigorously designed, prospective studies. In addition, the observed risk signals may be affected by reporting bias, including interference from factors such as media attention and changes in regulatory measures. Finally, some newly marketed ADCs lack long-term safety data, which limits our comprehensive understanding of their hematological toxicity characteristics.

## 5. Conclusion

This study systematically investigated the association between ADCs and hematotoxicity, identifying both established and new possible risk signals for hematotoxicity AEs. With the gradual introduction of ADCs to the market and their widespread clinical applications, enhancing pharmacovigilance efforts is essential. Future research should integrate information from FAERS and other pharmacovigilance databases to comprehensively assess and monitor ADCs-related hematotoxicity. In clinical practice, healthcare professionals must remain vigilant not only for AEs listed in the drug inserts but also for previously unreported hematological complications. Early detection and prompt intervention are crucial, and clinicians should promptly employ diagnostic, preventive, and therapeutic measures once an abnormality is detected. A deeper understanding of ADCs-related hematotoxicity is essential to optimize the safety profile of ADCs. Therefore, additional research is urgently needed to elucidate the mechanisms of hematotoxicity AEs and develop strategies to mitigate the associated risks.

## Supporting information

S1 Fig Results of subgroup disproportionate analyses of ADCs-related hematotoxicity based on age and sex (heatmap of EBGM05). ADCs, antibody-drug conjugates; EBGM05, lower limit of the 95% CI for the empirical Bayesian geometric mean.
(PDF)

S2 Fig Heatmap of EBGM05 based on the HLT classification of the ADCs. ADCs, antibody-drug conjugates; EBGM05, lower limit of the 95% CI for empirical Bayesian geometric mean; HLT, higher-level terms.
(PDF)

S1 Table The 2 × 2 conjunction tables for disproportionality analysis. (DOCX)

S2 Table Four major algorithms are used for signal detection. (DOCX)

S3 Table The clinical characteristics of cases with ADCs-related hematotoxicity from 2011Q3 to 2024Q2 within the FAERS database. (DOCX)

S4 Table ADCs-related hematotoxicity AEs signal intensity at preferred term level. (DOCX)

S5 Table The signal values of the top ten most common categories of ADCs-related hematotoxicity AEs according to different ADCs treatment regimens. (DOCX)
