# Supplementary material for: Hematological toxicity of anti-tumor antibody-drug conjugates: A retrospective pharmacovigilance study using the FDA adverse event reporting system
Source: PLoS One. 2025 Oct 27;20(10):e0334513. doi: 10.1371/journal.pone.0334513 (PMC12558476; doi:10.1371/journal.pone.0334513)

S1 Fig. Results of subgroup disproportionate analyses of ADCs-related hematotoxicity based on age and sex (heatmap of EBGM05). ADCs, antibody-drug conjugates; EBGM05, lower limit of the 95% CI for the empirical Bayesian geometric mean.

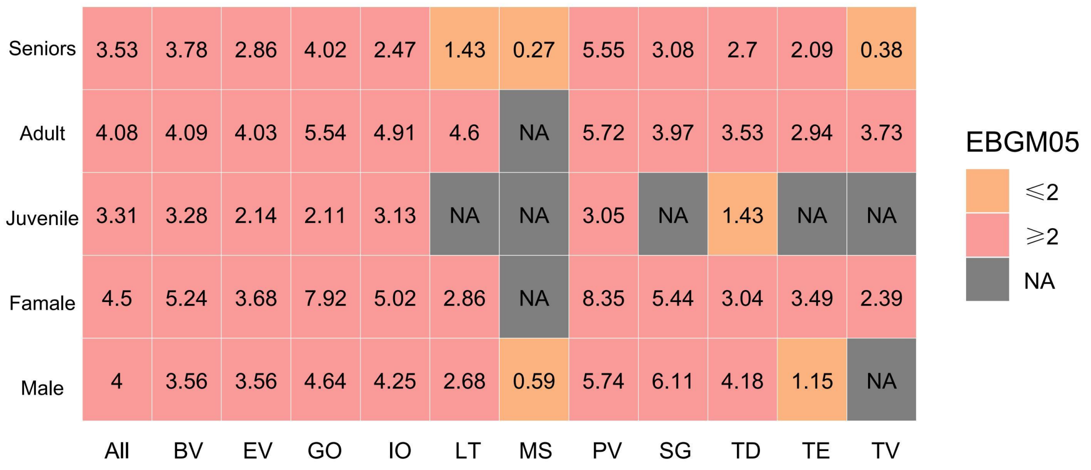

Supplement: S1 Fig — ADCs, antibody-drug conjugates; EBGM05, lower limit of the 95% CI for the empirical Bayesian geometric mean. (PDF) [file pone.0334513.s001.pdf]
