# Supplementary material for: Hematological toxicity of anti-tumor antibody-drug conjugates: A retrospective pharmacovigilance study using the FDA adverse event reporting system
Source: PLoS One. 2025 Oct 27;20(10):e0334513. doi: 10.1371/journal.pone.0334513 (PMC12558476; doi:10.1371/journal.pone.0334513)

S2 Fig. Heatmap of EBGM05 based on the HLT classification of the ADCs. ADCs, antibody-drug conjugates; EBGM05, lower limit of the 95% CI for empirical Bayesian geometric mean; HLT, higher-level terms.

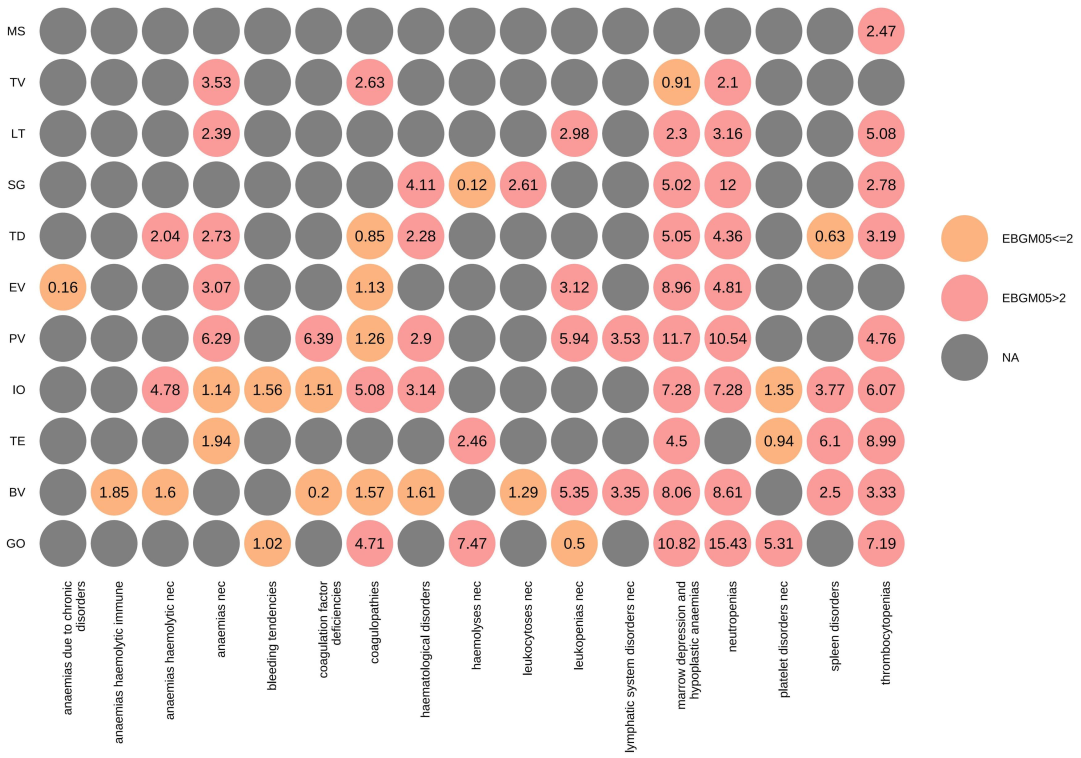

Supplement: S2 Fig — ADCs, antibody-drug conjugates; EBGM05, lower limit of the 95% CI for empirical Bayesian geometric mean; HLT, higher-level terms. (PDF) [file pone.0334513.s002.pdf]
