# Supplementary material for: Hematological toxicity of anti-tumor antibody-drug conjugates: A retrospective pharmacovigilance study using the FDA adverse event reporting system
Source: PLoS One. 2025 Oct 27;20(10):e0334513. doi: 10.1371/journal.pone.0334513 (PMC12558476; doi:10.1371/journal.pone.0334513)
Supplement: S1 Table — (DOCX) [file pone.0334513.s003.docx]

**S1 Table. The 2×2 conjunction tables for disproportionality analysis.**

|  | **Events for hematological toxicity** | **All other events** | **Total** |
| --- | --- | --- | --- |
| **Target ADCs** | a | b | a+b |
| **All other drugs** | c | d | c+d |
| **Total** | a+c | b+d | a+b+c+d |

Note: ADCs, antibody-drug conjugates; a, the number of hematological toxicity reports for ADCs; b, the number of reports for ADCs without hematological toxicity; c, the number of hematological toxicity reports for all other drugs; d, the number of reports for all other drugs without hematological toxicity.
