# Supplementary material for: Hematological toxicity of anti-tumor antibody-drug conjugates: A retrospective pharmacovigilance study using the FDA adverse event reporting system
Source: PLoS One. 2025 Oct 27;20(10):e0334513. doi: 10.1371/journal.pone.0334513 (PMC12558476; doi:10.1371/journal.pone.0334513)
Supplement: S2 Table — (DOCX) [file pone.0334513.s004.docx]

**S2 Table. The four major algorithms used for signal detection.**

| **Algorithms** | **Equation** | **Criteria** |
| --- | --- | --- |
| **ROR** | ROR=ad/b/c | lower limit of 95% CI > 1, N ≥ 3 |
|  | 95%CI=e^ln(R^°^R)±1.96(1/a+1/b+1/c+1/d)^0.5^ |  |
| **PRR** | PRR=a(c+d)/c/(a+b) | PRR ≥ 2, χ^2^ ≥ 4, N ≥ 3 |
|  | χ^2^=[(ad-bc)^2](a+b+c+d)/[(a+b)(c+d)(a+c)(b+d)] |  |
| **BCPNN** | IC=log_2_a(a+b+c+d)(a+c)(a+b) | IC025 > 0 |
|  | 95%CI= e^ln(IC)±1.96(1/a+1/b+1/c+1/d)^0.5^ |  |
| **MGPS** | EBGM=a(a+b+c+d)/(a+c)/(a+b) | EBGM05 > 2 |
|  | 95%CI=e^ln(EBGM)±1.96(1/a+1/b+1/c+1/d)^0.5^ |  |

Note: χ^2^, chi-square; BCPNN, bayesian confidence propagation neural network; CI, confidence interval; EBGM, empirical bayesian geometric mean; EBGM05, the lower limit of 95% CI of EBGM; IC, information component; IC025, the lower limit of 95% CI of IC; MGPS, multi-item gamma poisson shrinker; N, number of reports; PRR, proportional reporting ratio; ROR, reporting odds ratios.
