# Supplementary material for: Hematological toxicity of anti-tumor antibody-drug conjugates: A retrospective pharmacovigilance study using the FDA adverse event reporting system
Source: PLoS One. 2025 Oct 27;20(10):e0334513. doi: 10.1371/journal.pone.0334513 (PMC12558476; doi:10.1371/journal.pone.0334513)
Supplement: S3 Table — (DOCX) [file pone.0334513.s005.docx]

**S3 Table. The clinical characteristics of cases with ADCs-related hematotoxicity from 2011Q3 to 2024Q2 within the FAERS database.**

| **Clinical Characteristics** | **GO** | **BV** | **TE** | **IO** | **PV** | **EV** | **TD** | **SG** | **LT** | **TV** | **MS** | **Total** |
| --- | --- | --- | --- | --- | --- | --- | --- | --- | --- | --- | --- | --- |
| **Total cases** | 231 | 1,308 | 553 | 185 | 487 | 548 | 713 | 709 | 19 | 33 | 17 | 4,803 |
| **Sex** |  |  |  |  |  |  |  |  |  |  |  |  |
| **Female** | 79  (34.20%) | 437  (33.41%) | 493  (89.15%) | 71  (38.38%) | 199  (40.86%) | 102 (18.61%) | 483  (67.74%) | 613  (86.46%) | 6  (31.58%) | 29  (87.88%) | 9  (52.94%) | 2,521  (52.49%) |
| **Male** | 75  (32.47%) | 524  (40.06%) | 12  (2.17%) | 96  (51.89%) | 246  (50.51%) | 433 (79.01%) | 134  (18.79%) | 74  (10.44%) | 10  (52.63%) | 0  (0.00%) | 0  (0.00%) | 1,604  (33.40%) |
| **Unspecified** | 77  (33.33%) | 347  (26.53%) | 48  (8.68%) | 18  (9.73%) | 42  (8.62%) | 13  (2.37%) | 96  (13.46%) | 22  (3.10%) | 3  (15.79%) | 4  (12.12%) | 8  (47.06) | 678  (14.12%) |
| **Age** |  |  |  |  |  |  |  |  |  |  |  |  |
| **Juvenile (< 18)** | 11  (4.76%) | 46  (3.52%) | 0  (0.00%) | 31  (16.76%) | 10  (2.05%) | 19  (3.47%) | 5  (0.70%) | 0  (0.00%) | 0  (0.00%) | 0  (0.00%) | 0  (0.00%) | 122  (2.54%) |
| **Adult (18–65)** | 79  (34.20) | 488  (37.31%) | 294  (53.16%) | 97  (52.43%) | 92  (18.89%) | 90  (16.42%) | 244  (34.22%) | 290  (40.90%) | 6  (31.58%) | 19  (57.58%) | 0  (0.00%) | 1,699  (35.37%) |
| **Seniors (≥ 65)** | 43  (18.61%) | 258  (19.72%) | 84  (15.19%) | 31  (16.76%) | 278  (57.08%) | 319 (58.21%) | 169  (23.70%) | 120  (16.93%) | 6  (31.58%) | 1  (3.03) | 2 (11.76%) | 1,311  (27.30%) |
| **Unspecified** | 98  (42.42%) | 516  (39.45%) | 175  (31.65%) | 26  (14.05%) | 107  (21.97%) | 120 (21.90%) | 295  (41.37%) | 299  (42.17%) | 7  (36.84%) | 13  (39.39%) | 15 (88.24%) | 1,671  (34.79%) |
| **Median (IQR)** | 56  (35.5–66) | 54  (32–68) | 55  (48–64) | 35  (21–62) | 71  (63–77) | 72  (64–77) | 61  (51–70) | 57  (48.75–67) | 66  (54–72) | 49.5  (38.5–56) | / | 60  (47–72) |
| **Reporting Sources** | |  |  |  |  |  |  |  |  |  |  |  |
| **Physician** | 163  (70.56%) | 837  (63.99%) | 302  (54.61%) | 128  (69.19%) | 439  (90.14%) | 309 (56.39%) | 487  (68.30%) | 453  (63.89%) | 14  (73.68%) | 20  (60.61%) | 8  (47.06) | 3,160  (65.79%) |
| **Pharmacist** | 2  (0.87%) | 52  (3.98%) | 45  (8.14%) | 7  (3.78%) | 9  (1.85%) | 47  (8.58%) | 55  (7.71%) | 70  (9.87%) | 1  (5.26%) | 2  (6.06%) | 1  (5.88) | 291  (6.06%) |
| **Other healthcare providers** | 59  (25.54%) | 244  (18.65%) | 161  (29.11%) | 37  (20.00%) | 25  (5.13%) | 47  (8.58%) | 129  (18.09%) | 166  (23.41%) | 4  (21.05%) | 9  (27.27%) | 3 (17.65%) | 884  (18.41%) |
| **Customers** | 5  (2.16%) | 154  (11.77%) | 43  (7.78%) | 13  (7.03%) | 12  (2.46%) | 144 (26.28%) | 42  (5.89%) | 20  (2.82%) | 0  (0.00%) | 2  (6.06%) | 5 (29.41%) | 440  (9.16%) |
| **Unspecified** | 2  (0.87%) | 21  (1.61%) | 2  (0.36%) | 0  (0.00%) | 2  (0.41%) | 1  (0.18%) | 0  (0.00%) | 0  (0.00%) | 0  (0.00%) | 0  (0.00%) | 0  (0.00%) | 28  (0.58%) |
| **Indications** |  |  |  |  |  |  |  |  |  |  |  |  |
| **Lymphoma** | 0  (0.00%) | 1,195  (91.36%) | 0  (0.00%) | 7  (3.78%) | 453  (93.02%) | 0  (0.00%) | 0  (0.00%) | 1  (0.14%) | 17  (89.47%) | 0  (0.00%) | 0  (0.00%) | 1,673  (34.83%) |
| **Breast cancer** | 0  (0.00%) | 0  (0.00%) | 465  (84.09%) | 0  (0.00%) | 0  (0.00%) | 0  (0.00%) | 441  (61.85%) | 518  (73.06%) | 0  (0.00%) | 0  (0.00%) | 0  (0.00%) | 1,424  (29.65%) |
| **Bladder cancer** | 0  (0.00%) | 0  (0.00%) | 1  (0.18%) | 0  (0.00%) | 0  (0.00%) | 427  (77.92%) | 2  (0.28%) | 43  (6.06%) | 0  (0.00%) | 0  (0.00%) | 0  (0.00%) | 473  (9.85%) |
| **Leukemia** | 193  (83.55%) | 4  (0.31%) | 0  (0.00%) | 130  (70.27%) | 0  (0.00%) | 0  (0.00%) | 0  (0.00%) | 0  (0.00%) | 0  (0.00%) | 0  (0.00%) | 0  (0.00%) | 327  (6.81%) |
| **Gastric cancer** | 0  (0.00%) | 0  (0.00%) | 0  (0.00%) | 0  (0.00%) | 0  (0.00%) | 0  (0.00%) | 127  (17.81%) | 0  (0.00%) | 0  (0.00%) | 0  (0.00%) | 0  (0.00%) | 127  (2.64%) |
| **Others** | 5  (2.16%) | 32  (2.45%) | 26  (4.70%) | 6  (3.24%) | 2  (0.41%) | 54  (9.85%) | 85  (11.92%) | 59  (8.32%) | 0  (0.00%) | 23  (69.70%) | 5  (29.41%) | 297  (6.18%) |
| **Missing or unspecified** | 33  (14.29%) | 77  (5.89%) | 61  (11.03%) | 42  (22.70%) | 32  (6.57%) | 67  (12.23%) | 58  (8.13%) | 88  (12.41%) | 2  (10.53%) | 10  (30.30%) | 12  (70.59%) | 482  (10.04%) |
| **Reporting Countries** |  |  |  |  |  |  |  |  |  |  |  |  |
| **America** | 37  (16.02%) | 296  (22.63%) | 150  (27.12%) | 61  (32.97%) | 36  (7.39%) | 95  (17.34%) | 185  (25.95%) | 131  (18.48%) | 7  (36.84%) | 19  (57.58%) | 17  (100%) | 1,034  (21.53%) |
| **Japan** | 6  (2.60%) | 228  (17.43%) | 30  (5.42%) | 38  (20.54%) | 227  (46.61%) | 351 (64.05%) | 188  (26.37%) | 2  (0.28%) | 0  (0.00%) | 1  (3.03) | 0  (0.00%) | 1,071  (22.30%) |
| **France** | 39  (16.88%) | 134  (10.24%) | 26  (4.70%) | 7  (3.78%) | 8  (1.64%) | 41  (7.48%) | 73  (10.24%) | 187  (26.38%) | 0  (0.00%) | 1  (3.03) | 0  (0.00%) | 516  (10.74%) |
| **China** | 3  (1.30%) | 118  (9.02%) | 116  (20.98%) | 7  (3.78%) | 56  (11.50%) | 5  (0.91%) | 62  (8.70%) | 34  (4.80%) | 8  (42.11%) | 2  (6.06%) | 0  (0.00%) | 411  (8.56%) |
| **Germany** | 13  (5.63%) | 147  (11.24%) | 21  (3.80%) | 6  (3.24%) | 17  (3.49%) | 9  (1.64%) | 15  (2.10%) | 36  (5.08%) | 0  (0.00%) | 0  (0.00%) | 0  (0.00%) | 264  (5.50%) |
| **Canada** | 4  (1.73%) | 19  (1.45%) | 16  (2.89%) | 7  (3.78%) | 1  (0.21%) | 3  (0.55%) | 66  (9.26%) | 89  (12.55%) | 0  (0.00%) | 2  (6.06%) | 0  (0.00%) | 207  (4.31%) |
| **Italy** | 8  (3.46%) | 31  (2.37%) | 21 (3.80%) | 3  (1.62%) | 10  (2.05%) | 3  (0.55%) | 11  (1.54%) | 24  (3.39%) | 0  (0.00%) | 1  (3.03) | 0  (0.00%) | 112  (2.33%) |
| **United Kingdom** | 51  (22.08%) | 33  (2.52%) | 22  (3.98%) | 1  (0.54%) | 2  (0.41%) | 0  (0.00%) | 6  (0.84%) | 13  (1.83%) | 1  (5.26%) | 1  (3.03) | 0  (0.00%) | 130  (2.71%) |
| **Spain** | 1  (0.43%) | 24  (1.83%) | 11  (1.99%) | 3  (1.62%) | 15  (3.08%) | 6  (1.09%) | 5  (0.70%) | 20  (2.82%) | 0  (0.00%) | 0  (0.00%) | 0  (0.00%) | 85  (1.77%) |
| **Brazil** | 5  (2.16%) | 17  (1.30%) | 11  (1.99%) | 2  (1.08%) | 1  (0.21%) | 3  (0.55%) | 25  (3.51%) | 14  (1.97%) | 0  (0.00%) | 0  (0.00%) | 0  (0.00%) | 78  (1.62%) |
| **Others** | 64  (27.71%) | 248  (18.96%) | 129  (23.33%) | 50  (27.03%) | 112  (23.00%) | 32  (5.84%) | 77  (10.80%) | 159  (22.43%) | 3  (15.79%) | 6  (18.18%) | 0  (0.00%) | 880  (18.32%) |
| **Unspecified** | 0  (0.00%) | 13  (0.99%) | 0  (0.00%) | 0  (0.00%) | 2  (0.41%) | 0  (0.00%) | 0  (0.00%) | 0  (0.00%) | 0  (0.00%) | 0  (0.00%) | 0  (0.00%) | 15  (0.31%) |
| **Outcomes** |  |  |  |  |  |  |  |  |  |  |  |  |
| **Death** | 46  (19.91%) | 240  (18.35%) | 47  (8.50%) | 51  (27.57%) | 86  (17.66%) | 114 (20.80%) | 154  (21.60%) | 135  (19.04%) | 1  (5.26%) | 4  (12.12%) | 1  (5.88%) | 879  (18.30%) |
| **Life-threatening** | 33  (14.29%) | 84  (6.42%) | 23  (4.16%) | 6  (3.24%) | 21  (4.31%) | 39  (7.12%) | 41  (5.75%) | 54  (7.62%) | 0  (0.00%) | 0  (0.00) | 0  (0.00%) | 301  (6.27%) |
| **Hospitalization** | 74  (32.03%) | 587  (44.88%) | 145  (26.22%) | 56  (30.27%) | 129  (26.49%) | 103  (18.80%) | 146  (20.48%) | 191  (26.94%) | 10  (52.63%) | 17  (51.52%) | 1  (5.88%) | 1459  (30.28%) |
| **Disability** | 0  (0.00%) | 4  (0.31%) | 4  (0.72%) | 1  (0.54%) | 2  (0.41%) | 0  (0.00%) | 0  (0.00%) | 6  (0.85%) | 0  (0.00%) | 0  (0.00) | 0  (0.00%) | 17  (0.35%) |
| **Others** | 69  (29.87%) | 329  (25.15%) | 251  (45.39%) | 58  (31.35%) | 203  (41.68%) | 202  (36.86%) | 255  (35.76%) | 231  (32.58%) | 6  (31.57%) | 5  (15.15%) | 5  (26.32%) | 1614  (33.60%) |
| **Unspecified** | 9  (3.90%) | 64  (4.89%) | 83  (15.01%) | 13  (7.03%) | 46  (9.45%) | 90  (16.42%) | 117  (16.41%) | 92  (12.98%) | 2  (10.53%) | 7  (21.21%) | 10 (58.82%) | 533  (11.10%) |

Notes: ADCs, antibody-drug conjugates; BV, brentuximab vedotin; EV, enfortumab eedotin; GO, gemtuzumab ozogamicin; IO, inotuzumab ozogamicin; IQR, interquartile range; LT, loncastuximab tesirine; MS, mirvetuximab soravtansine; PV, polatuzumab vedotin; SG, sacituzumab govitecan; TD, trastuzumab deruxtecan; TE, trastuzumab emtansine; TV, tisotumab vedotin.
