# Supplementary material for: Hematological toxicity of anti-tumor antibody-drug conjugates: A retrospective pharmacovigilance study using the FDA adverse event reporting system
Source: PLoS One. 2025 Oct 27;20(10):e0334513. doi: 10.1371/journal.pone.0334513 (PMC12558476; doi:10.1371/journal.pone.0334513)
Supplement: S4 Table — (DOCX) [file pone.0334513.s006.docx]

**S4 Table. ADCs-related hematotoxicity AEs signal intensity at preferred term level.**

| **Drugname** | **Preferred term** | **Report number**  **(a)** | **ROR**  **(95%CI)** | **PRR**  **(χ^2^)** | **EBGM**  **(EBGM 05)** | **IC**  **(IC025)** |
| --- | --- | --- | --- | --- | --- | --- |
| **GO** |  |  |  |  |  |  |
|  | febrile neutropenia* | 124 | 54.63 (45.57–65.48) | 51.71 (6157.20) | 51.58 (44.32) | 5.69 (4.02) |
|  | thrombocytopenia* | 39 | 10.06 (7.33–13.80) | 9.9 (312.45) | 9.90 (7.59) | 3.31 (1.64) |
|  | bone marrow failure | 25 | 32.21 (21.71–47.78) | 31.86 (746.5) | 31.82 (22.87) | 4.99 (3.32) |
|  | neutropenia* | 22 | 4.29 (2.82–6.53) | 4.26 (54.94) | 4.26 (3.00) | 2.09 (0.42) |
|  | pancytopenia | 19 | 9.99 (6.36–15.7) | 9.92 (152.42) | 9.91 (6.79) | 3.31 (1.64) |
|  | cytopenia | 8 | 17.65 (8.81–35.34) | 17.59 (125.08) | 17.57 (9.83) | 4.14 (2.47) |
|  | disseminated intravascular coagulation | 7 | 16.27 (7.74–34.17) | 16.22 (99.92) | 16.21 (8.71) | 4.02 (2.35) |
|  | febrile bone marrow aplasia | 5 | 32.66 (13.57–78.59) | 32.59 (152.86) | 32.54 (15.60) | 5.02 (3.36) |
|  | hemolysis | 3 | 11.07 (3.57–34.36) | 11.06 (27.43) | 11.05 (4.28) | 3.47 (1.80) |
|  | coagulopathy | 3 | 5.27 (1.70–16.37) | 5.27 (10.37) | 5.27 (2.04) | 2.40 (0.73) |
|  | intravascular hemolysis | 2 | 120.03 (29.88–482.19) | 119.92 (234.49) | 119.23 (37.24) | 6.90 (5.22) |
|  | immune thrombocytopenia | 2 | 12.57 (3.14–50.30) | 12.56 (21.26) | 12.55 (3.93) | 3.65 (1.98) |
|  | platelet disorder | 2 | 20.8 (5.19–83.28) | 20.78 (37.62) | 20.76 (6.50) | 4.38 (2.71) |
|  | hemorrhagic diathesis | 2 | 15.13 (3.78–60.57) | 15.12 (26.35) | 15.11 (4.73) | 3.92 (2.25) |
|  | monocytopenia | 1 | 291.37 (40.46–2098.5) | 291.24 (285.17) | 287.16 (55.04) | 8.17 (6.46) |
| **BV** |  |  |  |  |  |  |
|  | febrile neutropenia * | 377 | 17.34 (15.65–19.21) | 17.05 (5657.17) | 16.92 (15.54) | 4.08 (2.41) |
|  | neutropenia * | 317 | 6.72 (6.01–7.51) | 6.64 (1516.14) | 6.62 (6.03) | 2.73 (1.06) |
|  | thrombocytopenia * | 150 | 4.14 (3.52–4.86) | 4.11 (353.43) | 4.11 (3.59) | 2.04 (0.37) |
|  | myelosuppression | 150 | 18.62 (15.85–21.88) | 18.49 (2462.41) | 18.35 (16.03) | 4.20 (2.53) |
|  | pancytopenia | 115 | 6.52 (5.43–7.84) | 6.49 (533.17) | 6.48 (5.55) | 2.70 (1.03) |
|  | leukopenia | 93 | 5.68 (4.63–6.96) | 5.66 (355.91) | 5.65 (4.76) | 2.50 (0.83) |
|  | lymphadenopathy * | 51 | 4.33 (3.29–5.71) | 4.33 (130.27) | 4.32 (3.43) | 2.11 (0.45) |
|  | lymphopenia * | 40 | 8.18 (6.00–11.17) | 8.17 (250.81) | 8.14 (6.28) | 3.03 (1.36) |
|  | bone marrow failure | 38 | 5.24 (3.81–7.20) | 5.23 (129.71) | 5.22 (4.00) | 2.38 (0.72) |
|  | febrile bone marrow aplasia | 34 | 24.17 (17.23–33.89) | 24.13 (745.65) | 23.88 (17.99) | 4.58 (2.91) |
|  | cytopenia | 28 | 6.66 (4.60–9.66) | 6.66 (134.17) | 6.64 (4.87) | 2.73 (1.06) |
|  | splenomegaly | 15 | 3.84 (2.31–6.37) | 3.84 (31.39) | 3.83 (2.51) | 1.94 (0.27) |
|  | disseminated intravascular coagulation | 14 | 3.50 (2.07–5.92) | 3.50 (25.00) | 3.50 (2.26) | 1.81 (0.14) |
|  | haematotoxicity | 11 | 3.56 (1.97–6.44) | 3.56 (20.24) | 3.56 (2.17) | 1.83 (0.16) |
|  | lymphadenitis | 5 | 6.32 (2.63–15.21) | 6.32 (22.33) | 6.30 (3.02) | 2.66 (0.99) |
|  | bicytopenia | 4 | 6.16 (2.31–16.43) | 6.16 (17.23) | 6.14 (2.7) | 2.62 (0.95) |
|  | hyperleukocytosis | 3 | 9.14 (2.94–28.41) | 9.14 (21.65) | 9.10 (3.53) | 3.19 (1.52) |
|  | abdominal lymphadenopathy | 3 | 8.99 (2.89–27.94) | 8.99 (21.21) | 8.96 (3.47) | 3.16 (1.49) |
|  | cold type haemolytic anaemia | 2 | 28.38 (7.03–114.5) | 28.38 (52.15) | 28.03 (8.72) | 4.81 (3.13) |
|  | leukostasis syndrome | 2 | 40.36 (9.97–163.44) | 40.35 (75.37) | 39.64 (12.3) | 5.31 (3.62) |
|  | bone marrow infiltration | 2 | 12.60 (3.14–50.60) | 12.60 (21.24) | 12.54 (3.92) | 3.65 (1.98) |
|  | coombs negative haemolytic anaemia | 2 | 32.11 (7.95–129.7) | 32.11 (59.41) | 31.66 (9.84) | 4.98 (3.30) |
|  | acquired von willebrand’s disease | 1 | 31.87 (4.43–229.53) | 31.87 (29.48) | 31.43 (6.03) | 4.97 (3.27) |
|  | splenic thrombosis | 1 | 15.60 (2.18–111.51) | 15.60 (13.57) | 15.49 (2.99) | 3.95 (2.27) |
|  | spleen congestion | 1 | 33.32 (4.62–240.11) | 33.32 (30.88) | 32.84 (6.29) | 5.04 (3.34) |
|  | hyposplenism | 1 | 75.84 (10.33–556.78) | 75.84 (71.39) | 73.34 (13.83) | 6.20 (4.45) |
|  | bone marrow granuloma | 1 | 45.82 (6.32–331.98) | 45.82 (42.95) | 44.90 (8.56) | 5.49 (3.77) |
| **TE** |  |  |  |  |  |  |
|  | thrombocytopenia * | 242 | 11.06 (9.74–12.57) | 10.87 (2166.93) | 10.84 (9.75) | 3.44 (1.77) |
|  | myelosuppression | 103 | 20.89 (17.20–25.37) | 20.73 (1923.73) | 20.62 (17.52) | 4.37 (2.70) |
|  | anemia * | 97 | 2.40 (1.97–2.93) | 2.39 (78.81) | 2.39 (2.02) | 1.26 (−0.41) |
|  | splenomegaly | 26 | 10.91 (7.42–16.04) | 10.89 (232.95) | 10.86 (7.87) | 3.44 (1.78) |
|  | cytopenia | 12 | 4.66 (2.65–8.22) | 4.66 (34.46) | 4.66 (2.90) | 2.22 (0.55) |
|  | hemolysis | 8 | 5.21 (2.61–10.43) | 5.21 (27.19) | 5.20 (2.91) | 2.38 (0.71) |
|  | thrombocytopenic purpura | 4 | 17.87 (6.69–47.73) | 17.86 (63.36) | 17.78 (7.81) | 4.15 (2.48) |
|  | hypersplenism | 2 | 20.75 (5.17–83.31) | 20.75 (37.37) | 20.63 (6.45) | 4.37 (2.69) |
|  | platelet anisocytosis | 1 | 257.12 (33.81–1955.52) | 257.1 (238.10) | 240.03 (43.95) | 7.91 (6.09) |
|  | splenic varices | 1 | 171.41 (23.05–1274.46) | 171.4 (161.71) | 163.65 (30.54) | 7.35 (5.58) |
|  | splenorenal shunt | 1 | 189.46 (25.36–1415.38) | 189.44 (178.08) | 180.02 (3.46) | 7.49 (5.71) |
| **IO** |  |  |  |  |  |  |
|  | febrile neutropenia* | 61 | 21.29 (16.51–27.44) | 20.84 (1152.12) | 20.82 (16.83) | 4.38 (2.71) |
|  | thrombocytopenia* | 41 | 8.65 (6.36–11.78) | 8.54 (273.25) | 8.54 (6.59) | 3.09 (1.43) |
|  | neutropenia* | 27 | 4.32 (2.96–6.31) | 4.29 (68.17) | 4.29 (3.12) | 2.10 (0.43) |
|  | pancytopenia* | 17 | 7.32 (4.54–11.79) | 7.28 (92.14) | 7.28 (4.88) | 2.86 (1.20) |
|  | cytopenia | 14 | 25.39 (15.01–42.95) | 25.27 (325.89) | 25.23 (16.25) | 4.66 (2.99) |
|  | myelosuppression* | 12 | 11.21 (6.36–19.77) | 11.17 (111.03) | 11.16 (6.94) | 3.48 (1.81) |
|  | thrombotic microangiopathy | 9 | 21.27 (11.05–40.95) | 21.21 (173.11) | 21.18 (12.25) | 4.40 (2.74) |
|  | bone marrow failure | 7 | 7.33 (3.49–15.39) | 7.31 (38.14) | 7.31 (3.93) | 2.87 (1.20) |
|  | haematotoxicity | 6 | 14.79 (6.64–32.97) | 14.76 (76.94) | 14.75 (7.55) | 3.88 (2.22) |
|  | splenic infarction | 4 | 55.65 (20.84–148.62) | 55.57 (213.64) | 55.39 (24.35) | 5.79 (4.12) |
|  | hemolytic anemia | 4 | 11.14 (4.18–29.72) | 11.13 (36.85) | 11.12 (4.89) | 3.48 (1.81) |
|  | disseminated intravascular coagulation | 3 | 5.71 (1.84–17.71) | 5.70 (11.63) | 5.70 (2.21) | 2.51 (0.84) |
|  | hemorrhagic diathesis* | 3 | 18.63 (6.00–57.84) | 18.61 (49.95) | 18.59 (7.21) | 4.22 (2.55) |
|  | anemia megaloblastic | 1 | 55.96 (7.85–398.72) | 55.94 (53.78) | 55.76 (10.78) | 5.80 (4.13) |
|  | acquired hemophilia | 1 | 21.65 (3.04–153.92) | 21.64 (19.66) | 21.61 (4.19) | 4.43 (2.76) |
|  | antiphospholipid syndrome | 1 | 14.14 (1.99–100.53) | 14.14 (12.20) | 14.13 (2.74) | 3.82 (2.15) |
|  | spleen disorder | 1 | 13.22 (1.86–93.93) | 13.21 (11.28) | 13.2 (2.56) | 3.72 (2.05) |
|  | platelet dysfunction | 1 | 66.40 (9.31–473.38) | 66.37 (64.13) | 66.12 (12.78) | 6.05 (4.37) |
| **PV** |  |  |  |  |  |  |
|  | anemia* | 144 | 7.86 (6.66–9.28) | 7.70 (840.96) | 7.69 (6.70) | 2.94 (1.28) |
|  | febrile neutropenia* | 129 | 21.01 (17.64–25.02) | 20.58 (2399.22) | 20.53 (17.74) | 4.36 (2.69) |
|  | neutropenia* | 112 | 8.44 (7.00–10.17) | 8.30 (719.64) | 8.29 (7.09) | 3.05 (1.38) |
|  | myelosuppression* | 70 | 30.82 (24.34–39.02) | 30.47 (1987.94) | 30.35 (24.91) | 4.92 (3.26) |
|  | thrombocytopenia* | 67 | 6.57 (5.16–8.36) | 6.51 (312.63) | 6.50 (5.32) | 2.70 (1.03) |
|  | cytopenia | 56 | 47.78 (36.7–62.21) | 47.34 (2525.42) | 47.06 (37.73) | 5.56 (3.89) |
|  | leukopenia* | 37 | 8.01 (5.80–11.07) | 7.97 (225.42) | 7.96 (6.07) | 2.99 (1.33) |
|  | pancytopenia* | 27 | 5.41 (3.71–7.90) | 5.39 (96.62) | 5.39 (3.93) | 2.43 (0.76) |
|  | agranulocytosis | 15 | 9.21 (5.55–15.30) | 9.19 (109.42) | 9.18 (6.01) | 3.20 (1.53) |
|  | lymphadenopathy | 13 | 3.91 (2.27–6.74) | 3.91 (28.10) | 3.90 (2.48) | 1.96 (0.30) |
|  | lymphopenia* | 9 | 6.51 (3.38–12.52) | 6.50 (41.85) | 6.49 (3.76) | 2.70 (1.03) |
|  | disseminated intravascular coagulation | 8 | 7.10 (3.55–14.21) | 7.09 (41.83) | 7.09 (3.97) | 2.83 (1.16) |
|  | haematotoxicity | 7 | 8.04 (3.83–16.88) | 8.03 (43.06) | 8.03 (4.31) | 3.00 (1.34) |
|  | abdominal lymphadenopathy | 5 | 53.31 (22.12–128.52) | 53.27 (254.7) | 52.91 (25.34) | 5.73 (4.06) |
|  | hypofibrinogenemia | 4 | 37.25 (13.94–99.51) | 37.22 (140.34) | 37.05 (16.28) | 5.21 (3.54) |
|  | bone marrow disorder | 2 | 7.30 (1.83–29.23) | 7.30 (10.87) | 7.30 (2.29) | 2.87 (1.20) |
|  | retroperitoneal lymphadenopathy | 2 | 37.69 (9.39–151.23) | 37.67 (71.06) | 37.5 (11.72) | 5.23 (3.56) |
|  | bone marrow infiltration | 1 | 22.28 (3.13–158.68) | 22.28 (20.27) | 22.22 (4.30) | 4.47 (2.80) |
|  | hilar lymphadenopathy | 1 | 12.44 (1.75–88.47) | 12.44 (10.50) | 12.42 (2.41) | 3.63 (1.96) |
| **EV** |  |  |  |  |  |  |
|  | myelosuppression | 156 | 39.25 (33.49–46.00) | 38.68 (5678.75) | 38.35 (33.58) | 5.26 (3.6) |
|  | anemia * | 121 | 3.70 (3.09–4.42) | 3.67 (235.20) | 3.66 (3.15) | 1.87 (0.21) |
|  | neutropenia | 111 | 4.70 (3.9–5.67) | 4.66 (319.70) | 4.66 (3.98) | 2.22 (0.55) |
|  | febrile neutropenia * | 96 | 8.75 (7.15–10.70) | 8.68 (651.49) | 8.66 (7.32) | 3.11 (1.45) |
|  | leukopenia | 39 | 4.78 (3.49–6.54) | 4.76 (115.88) | 4.76 (3.66) | 2.25 (0.58) |
|  | bone marrow failure | 12 | 3.32 (1.88–5.84) | 3.31 (19.39) | 3.31 (2.06) | 1.73 (0.06) |
|  | disseminated intravascular coagulation* | 12 | 6.04 (3.43–10.64) | 6.03 (50.34) | 6.03 (3.75) | 2.59 (0.93) |
|  | cytopenia | 11 | 5.25 (2.91–9.49) | 5.25 (37.77) | 5.24 (3.20) | 2.39 (0.72) |
|  | blood loss anemia | 4 | 10.30 (3.86–27.49) | 10.30 (33.51) | 10.28 (4.52) | 3.36 (1.69) |
|  | anemia of malignant disease | 1 | 19.65 (2.76–140.14) | 19.65 (17.62) | 19.57 (3.78) | 4.29 (2.61) |
| **TD** |  |  |  |  |  |  |
|  | neutropenia | 178 | 4.78 (4.12–5.54) | 4.74 (525.19) | 4.73 (4.18) | 2.24 (0.58) |
|  | anemia* | 170 | 3.29 (2.83–3.82) | 3.26 (267.42) | 3.26 (2.87) | 1.71 (0.04) |
|  | thrombocytopenia | 112 | 3.93 (3.26–4.73) | 3.91 (242.51) | 3.90 (3.34) | 1.97 (0.31) |
|  | myelosuppression | 111 | 17.5 (14.51–21.1) | 17.39 (1704.35) | 17.29 (14.78) | 4.11 (2.45) |
|  | febrile neutropenia* | 110 | 6.33 (5.25–7.64) | 6.30 (489.71) | 6.29 (5.37) | 2.65 (0.99) |
|  | pancytopenia | 53 | 3.81 (2.91–4.99) | 3.80 (109.46) | 3.80 (3.03) | 1.93 (0.26) |
|  | haematotoxicity | 22 | 9.10 (5.98–13.83) | 9.08 (157.8) | 9.06 (6.38) | 3.18 (1.51) |
|  | cytopenia | 19 | 5.75 (3.66–9.02) | 5.74 (74.31) | 5.73 (3.93) | 2.52 (0.85) |
|  | disseminated intravascular coagulation* | 14 | 4.46 (2.64–7.54) | 4.46 (37.52) | 4.45 (2.87) | 2.16 (0.49) |
|  | hemolytic anemia | 8 | 3.73 (1.86–7.46) | 3.73 (15.95) | 3.72 (2.08) | 1.90 (0.23) |
|  | immune thrombocytopenia | 7 | 6.05 (2.88–12.7) | 6.05 (29.42) | 6.04 (3.24) | 2.59 (0.93) |
|  | splenic embolism | 2 | 62.23 (15.33–252.7) | 62.23 (117.86) | 60.89 (18.85) | 5.93 (4.24) |
| **SG** |  |  |  |  |  |  |
|  | neutropenia * | 356 | 14.01 (12.61–15.57) | 13.61 (4155.31) | 13.57 (12.42) | 3.76 (2.10) |
|  | febrile neutropenia* | 178 | 14.85 (12.80–17.22) | 14.64 (2255.68) | 14.59 (12.88) | 3.87 (2.20) |
|  | thrombocytopenia* | 75 | 3.77 (3.00–4.73) | 3.75 (151.50) | 3.75 (3.10) | 1.91 (0.24) |
|  | pancytopenia | 44 | 4.54 (3.38–6.11) | 4.53 (120.88) | 4.52 (3.53) | 2.18 (0.51) |
|  | myelosuppression | 35 | 7.85 (5.63–10.94) | 7.83 (208.15) | 7.82 (5.92) | 2.97 (1.30) |
|  | leukopenia | 32 | 3.56 (2.51–5.03) | 3.55 (58.55) | 3.55 (2.65) | 1.83 (0.16) |
|  | febrile bone marrow aplasia | 28 | 36.30 (25.01–52.68) | 36.21 (950.21) | 35.9 (26.28) | 5.17 (3.50) |
|  | haematotoxicity | 20 | 11.86 (7.64–18.40) | 11.84 (197.94) | 11.81 (8.18) | 3.56 (1.90) |
|  | cytopenia | 17 | 7.38 (4.58–11.88) | 7.37 (93.43) | 7.36 (4.94) | 2.88 (1.21) |
|  | agranulocytosis | 14 | 4.42 (2.62–7.47) | 4.42 (36.99) | 4.41 (2.85) | 2.14 (0.48) |
|  | blood disorder | 6 | 4.01 (1.80–8.93) | 4.01 (13.53) | 4.00 (2.05) | 2.00 (0.34) |
|  | bone marrow infiltration | 1 | 11.47 (1.61–81.69) | 11.47 (9.53) | 11.44 (2.21) | 3.52 (1.84) |
|  | hemoglobinemia | 1 | 30.42 (4.25–217.62) | 30.42 (28.24) | 30.20 (5.82) | 4.92 (3.23) |
| **LT** |  |  |  |  |  |  |
|  | thrombocytopenia* | 7 | 10.78 (5.10–22.77) | 10.60 (60.98) | 10.60 (5.67) | 3.41 (1.73) |
|  | anemia* | 6 | 5.06 (2.26–11.34) | 5.00 (19.26) | 5.00 (2.55) | 2.32 (0.65) |
|  | neutropenia* | 6 | 7.02 (3.14–15.73) | 6.93 (30.51) | 6.93 (3.53) | 2.79 (1.12) |
|  | leukopenia | 3 | 10.14 (3.26–31.59) | 10.07 (24.53) | 10.07 (3.89) | 3.33 (1.66) |
|  | cytopenia | 1 | 13.14 (1.85–93.57) | 13.11 (11.19) | 13.11 (2.54) | 3.71 (2.04) |
| **TV** |  |  |  |  |  |  |
|  | anemia | 17 | 5.70 (3.53–9.20) | 5.61 (64.66) | 5.61 (3.76) | 2.49 (0.82) |
|  | febrile neutropenia | 8 | 7.93 (3.95–15.90) | 7.87 (48.03) | 7.87 (4.40) | 2.98 (1.31) |
|  | thrombotic microangiopathy | 2 | 13.6 (3.40–54.47) | 13.57 (23.29) | 13.57 (4.25) | 3.76 (2.09) |
|  | cytopenia | 2 | 10.41 (2.60–41.7) | 10.39 (16.98) | 10.39 (3.25) | 3.38 (1.71) |
| **MS** |  |  |  |  |  |  |
|  | thrombocytopenia* | 9 | 4.81 (2.50–9.27) | 4.78 (26.92) | 4.78 (2.76) | 2.26 (0.59) |

Notes: χ^2^, chi-square; ADCs, antibody-drug conjugates; AE, adverse event; BV, brentuximab vedotin; CI, confidence interval; EBGM, empirical bayesian geometric mean; EBGM05, the lower limit of 95% CI of EBGM; EV, enfortumab vedotin; GO, gemtuzumab ozogamicin; IC, information component; IC025, the lower limit of 95% CI of IC; IO, inotuzumab ozogamicin; LT, loncastuximab tesirine; MS, mirvetuximab soravtansine; PRR, proportional reporting ratio; PT, preferred term; PV, polatuzumab vedotin; ROR, reporting odds ratios; SG, sacituzumab govitecan; TD, trastuzumab deruxtecan; TE, trastuzumab emtansine; TV, tisotumab vedotin.

* Adverse reactions mentioned in the drug label.
