# Supplementary material for: Hematological toxicity of anti-tumor antibody-drug conjugates: A retrospective pharmacovigilance study using the FDA adverse event reporting system
Source: PLoS One. 2025 Oct 27;20(10):e0334513. doi: 10.1371/journal.pone.0334513 (PMC12558476; doi:10.1371/journal.pone.0334513)
Supplement: S5 Table — (DOCX) [file pone.0334513.s007.docx]

**S5 Table. The signal values of the top ten most common categories of ADCs-related hematotoxicity AEs according to different ADCs treatment regimens.**

| **PT** | **ROR (95% CI)** | **PRR (χ^2^)** | **EBGM (EBGM 05)** | **IC (IC 025)** |
| --- | --- | --- | --- | --- |
| **Neutropenia** | 6.29 (5.94–6.67) | 6.22 (5,089.33) | 6.16 (5.87) | 2.62 (0.96) |
| **Febrile neutropenia** | 11.49 (10.78–12.24) | 11.37 (9,153.82) | 11.16 (10.58) | 3.48 (1.81) |
| **Anaemia** | 3.37 (3.15–3.60) | 3.35 (1,439.68) | 3.33 (3.15) | 1.74 (0.07) |
| **Thrombocytopenia** | 5.31 (4.95–5.71) | 5.27 (2,609.04) | 5.23 (4.93) | 2.39 (0.72) |
| **Myelosuppression** | 20.52 (18.96–22.21) | 20.37 (11,357.3) | 19.68 (18.42) | 4.30 (2.63) |
| **Pancytopenia** | 4.23 (3.78–4.75) | 4.22 (722.93) | 4.2 (3.81) | 2.07 (0.40) |
| **Leukopenia** | 3.83 (3.38–4.34) | 3.83 (516.53) | 3.81 (3.43) | 1.93 (0.26) |
| **Cytopenia** | 9.72 (8.31–11.37) | 9.70 (1,227.61) | 9.55 (8.38) | 3.26 (1.59) |
| **Lymphadenopathy** | 2.14 (1.76–2.60) | 2.14 (60.35) | 2.13 (1.81) | 1.09 (−0.57) |
| **Bone marrow failure** | 2.95 (2.39–3.65) | 2.95 (109.05) | 2.94 (2.46) | 1.56 (−0.11) |

Notes: ADCs, antibody-drug conjugates; PT, preferred term; ROR, reporting odds ratio; CI, confidence interval; PRR, proportional reporting ratio; EBGM, empirical Bayesian geometric mean; IC, information component.
